# Semi-automatic quantification of 4D left ventricular blood flow

**DOI:** 10.1186/1532-429X-12-9

**Published:** 2010-02-12

**Authors:** Jonatan Eriksson, Carl Johan Carlhäll, Petter Dyverfeldt, Jan Engvall, Ann F Bolger, Tino Ebbers

**Affiliations:** 1 Division of Cardiovascular Medicine, Department of Medical and Health Sciences, Linköping University, Linköping, Sweden; 2 Center for Medical Image Science and Visualization (CMIV), Linköping University, Linköping, Sweden; 3 Department of Clinical Physiology, Linköping University Hospital, Linköping, Sweden; 4 Division of Applied Thermodynamics and Fluid Mechanics, Department of Management and Engineering, Linköping University, Linköping, Sweden; 5 Department of Medicine, University of California, San Francisco, California, USA

## Abstract

**Background:**

The beating heart is the generator of blood flow through the cardiovascular system. Within the heart's own chambers, normal complex blood flow patterns can be disturbed by diseases. Methods for the quantification of intra-cardiac blood flow, with its 4D (3D+time) nature, are lacking. We sought to develop and validate a novel semi-automatic analysis approach that integrates flow and morphological data.

**Method:**

In six healthy subjects and three patients with dilated cardiomyopathy, three-directional, three-dimensional cine phase-contrast cardiovascular magnetic resonance (CMR) velocity data and balanced steady-state free-precession long- and short-axis images were acquired. The LV endocardium was segmented from the short-axis images at the times of isovolumetric contraction (IVC) and isovolumetric relaxation (IVR). At the time of IVC, pathlines were emitted from the IVC LV blood volume and traced forwards and backwards in time until IVR, thus including the entire cardiac cycle. The IVR volume was used to determine if and where the pathlines left the LV. This information was used to automatically separate the pathlines into four different components of flow: *Direct Flow*, *Retained Inflow*, *Delayed Ejection Flow *and *Residual Volume*. Blood volumes were calculated for every component by multiplying the number of pathlines with the blood volume represented by each pathline. The accuracy and inter- and intra-observer reproducibility of the approach were evaluated by analyzing volumes of LV inflow and outflow, the four flow components, and the end-diastolic volume.

**Results:**

The volume and distribution of the LV flow components were determined in all subjects. The calculated LV outflow volumes [ml] (67 ± 13) appeared to fall in between those obtained by through-plane phase-contrast CMR (77 ± 16) and Doppler ultrasound (58 ± 10), respectively. Calculated volumes of LV inflow (68 ± 11) and outflow (67 ± 13) were well matched (NS). Low inter- and intra-observer variability for the assessment of the volumes of the flow components was obtained.

**Conclusions:**

This semi-automatic analysis approach for the quantification of 4D blood flow resulted in accurate LV inflow and outflow volumes and a high reproducibility for the assessment of LV flow components.

## Introduction

The final product of the molecular, electrical and mechanical events in the normal heart is the generation of blood flow. The forces which result from interaction between the heart and the flowing blood stimulate a continuous remodeling process [1], interactively creating an optimal geometry for efficient flow. Alterations in left ventricular (LV) flow patterns have been recognized in various cardiac diseases such as LV wall motion disorders, valvular disease and arrhythmia. In heart failure, the LV may undergo progressive adverse remodeling [2]. In these hearts, abnormal LV blood flow patterns have been observed [3-5]. These altered flow patterns may be detrimental to LV function, and in a vicious cycle, contribute further to the adverse remodeling. Thus, it is desirable to gain a deeper understanding of the LV blood flow behavior under normal and disordered conditions.

Previous methods have provided information about some aspects of LV blood flow behavior. These methods have been limited in their ability to assess the time-varying and intrinsically three-dimensional (time + 3D = 4D) flow patterns within the beating LV. Limitations have arisen due to velocity being recorded from single directional velocity components (as with Doppler ultrasound) [6] or from two-dimensional (2D) regions of interest with cardiovascular magnetic resonance (CMR) [7,8]. Computational fluid dynamics (CFD) of the heart is being used more frequently [9-12] and has the potential to provide relevant information in the future. CFD may produce data with higher spatial and temporal resolution than actual in vivo data and creates the opportunity to broadly examine different measures of flow and the impact of varying conditions. However, today, the complex blood flow patterns in the heart are difficult to simulate accurately. Three-dimensional cine phase-contrast CMR (3D cine PC-CMR) has the ability to provide measurements of a time-varying velocity field, which allows for streamline [13] and pathline visualization [14-17] of the blood flow patterns over a complete cardiac cycle. This method has been applied most frequently to the aorta [18-20], but a few studies have also been performed in the beating heart [21,22].

Recently, we have developed CMR based tools that elucidate 4D LV blood flow patterns [22]. This technique enables the quantification of the volume, distribution and kinetic energy change of separate LV flow components over the cardiac cycle. In order to reduce user-dependency and enhance the reproducibility of this technique, we sought to develop and validate a semi-automatic analysis approach that better integrates flow and morphological data.

## Method

### Measurements

Nine subjects were included in the study, mean age 54 years (range 22-62); six healthy subjects (three females), mean age 58 (range 50-61) years, and three patients (one female) suffering from idiopathic dilated cardiomyopathy (DCM), mean age 46 (range 22-62) years (Table 1). The healthy subjects had normal electrocardiograms and echocardiographic examinations without valvular or ventricular dysfunction. No patient had more than trace valvular insufficiency or stenosis. The study was approved by the regional Ethical Review Board in Linköping, Sweden. All nine subjects gave written informed consent before participation.

**Table 1:** Demographic and clinical data

	Healthy subjects (n = 6)	DCM (n = 3)
Age (yrs)	58 (range 50-61)	46 (range 22-62)
Gender (f:m)	3:3	1:2
Weight (kg)	72 ± 6	78 ± 18
Heart rate (bpm)	64 ± 8	60 ± 17
Systolic BP (mm Hg)	128 ± 10	120 ± 17
Diastolic BP (mm Hg)	80 ± 5	78 ± 8
LVEDD (mm)	45 ± 3	60 ± 4
LVEF (%)	59 ± 2	39 ± 4

Mean ± SD. BP, blood pressure; DCM, dilated cardiomyopathy; EDD, end-diastolic diameter; EF, ejection fraction; LV, left ventricle.

In all nine subjects, 3D cine PC-CMR velocity data of the left heart, morphological long- and short-axis data of the LV, and 2D cine through-plane PC-CMR velocity data of the ascending aorta were acquired on a clinical 1.5 T scanner (Philips Achieva, Philips Medical Systems, Best, the Netherlands). Echocardiography was performed using a Vivid 7 scanner and a 2.0 MHz probe (GE, Vingmed Ultrasound, Horten, Norway).

Cine balanced steady-state free-precession (bSSFP) imaging was used to acquire 2-, 3- and 4-chamber long-axis and a stack of short-axis morphological images in 30 time frames during end-expiratory breath holds. A slice thickness of 8 mm was used. Acquired pixel size was 2.19 × 1.78 mm^2 ^and 1.67 × 1.78 mm^2 ^for the short- and long-axis images, respectively. Reconstructed pixel size was of 1.25^2 ^mm^2 ^for the long-axis images and 1.37^2 ^mm^2 ^for the short-axis images.

Two-dimensional cine through-plane PC-CMR velocity data were acquired in a plane perpendicular to the main flow direction in the ascending aorta (AoA) just downstream from the aortic valve (AoV). Imaging parameters included a velocity encoding range (VENC) of 200 cm/s, an echo time (TE) of 3 ms, a repetition time (TR) of 5 ms, a flip angle of 15°, a slice thickness of 7 mm, and a pixel size of 1.6 × 1.6 mm^2^. Three lines of k-space were acquired per heart beat, resulting in a temporal resolution of 30 ms.

The three-directional, 3D cine PC-CMR velocity data were acquired during free-breathing, using a navigator-gated gradient-echo pulse-sequence with interleaved three-directional flow-encoding and retrospective, vector cardiogram controlled cardiac gating [23]. Common acquisition parameters included a VENC of 100 cm/s, a flip angle of 8°, a TE of 3.7 ms, and a TR of 6.3 ms. Parallel imaging by sensitivity encoding (SENSE) with a speed-up factor of 2 was applied. To further reduce the scan time, two k-space lines per flow encoding segment were acquired during each heart beat. The spatial resolution was 3 × 3 × 3 mm^3^. The field-of-view (FOV) was adjusted for each subject to fully encompass the left heart. Scan time was about 10-15 minutes, excluding the navigator gating efficiency. A temporal sliding-window reconstruction with individual non-linear stretching of each RR-interval (Philips Medical Systems, Best, The Netherlands) was used to reconstruct the time-resolved PC-CMR data into 40 time frames. Data were corrected for concomitant gradient field effects on the scanner. By the use of automated in-house postprocessing software written in Matlab (The MathWorks Inc., Natick, Massachusetts, USA), the velocity data were corrected for background errors and phase wraps and converted into a file format compatible with commercially available visualization software (EnSight, CEI Inc, Research Triangle Park, NC, USA). The background correction scheme, which was performed for each time frame, consisted of a 4^th^-order weighted least-squares fit to the static tissue of the measured velocity data, where the weighting function was obtained from a combination of the signal magnitude and the standard deviation of the measured velocity over time [24].

After postprocessing, all 3D cine PC-CMR datasets underwent quality control, which consisted of visual inspection of pathlines emitted from a plane located approximately at the mitral valve (MV) annulus and integrated over one cardiac cycle. In addition to being valuable for visualization and quantification, pathlines provide a means of performing internal control of the data quality. As pathlines are calculated by the integration of the time-resolved velocity field, a pathline should never leave the blood pool. Although in some cases a few pathlines may leave the blood pool due to noise, it is important to make sure that there are no systematic velocity offsets in the data: such an offset may result in a set of aberrant pathlines leaving the blood pool at a specific site. With our present data acquisition and postprocessing protocol, the vast majority of our data sets pass this quality control.

### Data analysis

The method proposed for the quantification of LV flow components consists of three steps:

1) Definition of geometrical constraints of the LV at the times of isovolumetric contraction (IVC) and isovolumetric relaxation (IVR).

2) Computation of pathlines, backwards and forwards in time, from the entire LV at the time of IVC.

3) Separation of the pathlines into different components using the IVR LV constraint.

The manual steps of this approach are the definition of geometrical constraints and the determination of the time of IVC and IVR in the 3D cine PC-CMR data. Pathlines calculation and separation of the pathlines into different components are fully automatic.

Following a pre-established protocol, the LV was segmented from the stack of short-axis images, to define geometrical constraints. The endocardial border was defined according to the limit of the compact myocardium, such that LV trabeculations were included in the estimated LV blood volume. The IVC and IVR phases were determined by visual inspection of the open or closed positions of the aortic and mitral valves and the LV size in the long- and short-axis images. The segmentation was performed at IVC and IVR using freely available segmentation software (Segment, version 1.699d) [25]. From the segmentations two binary masks were created, each consisting of a stack of 8 mm thick slices. These were used to create two masks of the LV volume that matched the orientation of the 3D cine PC-CMR data. The IVC LV volume was resampled to match the 3D cine PC-CMR data resolution, using in-house developed Matlab software and was considered to contain all the blood residing in the LV during the analyzed cardiac cycle.

Pathlines were emitted from the resampled IVC LV volume at the time of IVC, estimated from the 3D cine PC-CMR data. In this method, the visual inspection of short streamlines emitted from a three-chamber plane was used to determine IVC and IVR as the time when no flow can be seen across either the mitral or aortic valve. The pathlines were emitted from the center point of each voxel in the IVC LV volume and traced both forward and backward in time until the time of IVR, thus covering the complete cardiac cycle. No mask was used in the pathline computation; the pathlines were allowed to go anywhere in the 3D cine PC-CMR dataset covering the whole heart. The computation of pathlines was performed in EnSight (version 8.2), which utilizes a 4^th ^order Runge-Kutta numerical integration technique with adaptive step length.

The pathlines, describing all the blood transiting the LV over a cardiac cycle, were analyzed using an automatic algorithm, which works as follows. The IVR LV volume was used to determine whether and where the pathlines left the LV. A pathline leaving the IVR LV volume through the basal plane was considered to have left the LV in the physiological way, i.e. through the AoV during systole (forward pathlines) or MV during diastole (backward pathlines). Pathlines remaining below the basal plane at the time for IVR were divided in two categories: pathlines remaining inside the confines of the IVR LV volume were considered to represent the LV blood, whereas pathlines extending outside of this volume were considered to either have been emitted from the myocardium or to have left the LV due to data imperfections, such as noise or uncompensated background phase errors. The second category of pathlines was excluded from further calculations.

Based on this combination of pathlines and geometrical constraints, the intraventricular blood flow was automatically divided into the four components, as defined by Bolger et al [22]:

• *Direct Flow*: Blood that enters the LV during diastole and leaves the LV during systole in the analyzed heart beat

• *Retained Inflow*: Blood that enters the LV during diastole but does not leave during systole in the analyzed heart beat

• *Delayed Ejection Flow*: Blood that starts and resides inside the LV during diastole and leaves during systole

• *Residual Volume*: Blood that resides within the LV for at least two cardiac cycles

Each of these pathlines was considered to represent a volume of blood defined by the emitter density, here chosen to correspond to the voxel size of the 3D cine PC-CMR data. The volumes of the different components were calculated by multiplying the number of pathlines in each component group with the voxel volume.

### Evaluation and statistical analysis

The analysis approach proposed was evaluated for accuracy as well as inter- and intra-observer variability on nine subjects. Accuracy was evaluated by comparing the LV inflow and outflow, where *Inflow = Direct Flow *+ *Retained Inflow *and *Outflow = Direct Flow *+ *Delayed Ejection Flow*. In addition, the outflow obtained by the proposed approach was related to measurements obtained by 2D cine through-plane PC-CMR and Doppler ultrasound, respectively.

Two investigators, referred to as Investigator 1 (Inv 1) and Investigator 2 (Inv 2), participated in the inter-observer study and carried out the analysis of all nine subjects independently of each other. Inv 1 had little experience and Inv 2 had moderate experience in segmenting the LV from short axis CMR images. To assess the intra-observer variability, Inv 1 repeated the analysis on all nine data sets approximately one month after the first analysis. The results from the first analysis of Inv 1 are referred to as Analysis 1 (A1) and the second as Analysis 2 (A2).

All values are given as group mean ± 1SD unless otherwise specified. In the statistical analyses, all nine subjects are considered as one group. For LV inflow versus outflow comparison, two-tailed paired t-tests were used, and the statistical significance was set at P < 0.05. In the observer variability studies, two-tailed paired t-tests were used to test for differences between the two investigators, or between Inv 1 A1 and Inv 1 A2. As five interrelated parameters were compared, a Bonferroni correction was performed, and the level of significance was adjusted to P < 0.01.

## Results

All nine datasets were acquired successfully. No significant aberrant traces, indicating artifacts or insufficient correction for background offset, were observed in any of the datasets.

**Figure 1:**
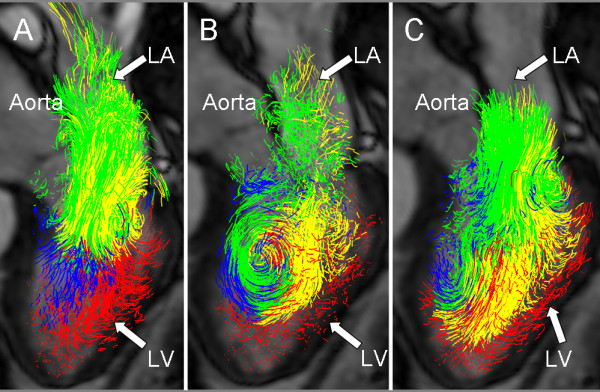
**Diastolic blood flow**. Pathline visualization of all blood flow involved in one cardiac cycle in the left ventricle (LV) of a healthy, 61 year old male at peak early LV filling (A), diastasis (B), and peak atrial contraction (C). In all panels a semitransparent three-chamber image provides anatomical orientation. The pathlines are color coded according to: *Direct Flow*, green; *Retained Inflow*, yellow; *Delayed Ejection Flow*, blue; *Residual Volume*, red. LA, Left Atrium; LV, Left Ventricle.

**Figure 2:**
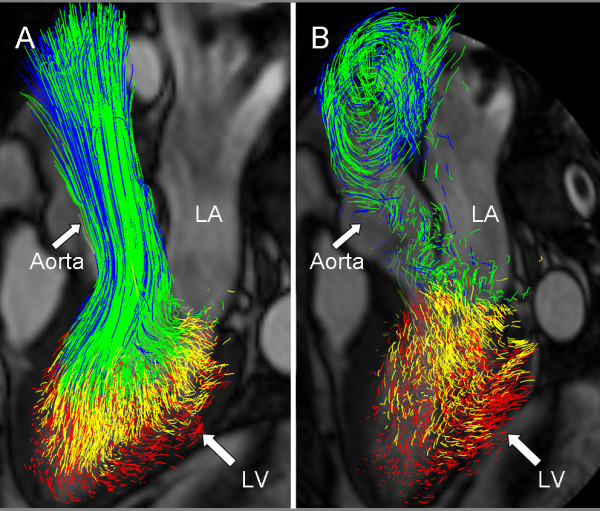
**Systolic blood flow**. Pathline visualization of all blood flow involved in one cardiac cycle in the left ventricle of a healthy, 61 year old male at peak systole (A), and at isovolumetric relaxation (B). In all panels a semitransparent three-chamber image provides anatomical orientation. The pathlines are color coded according to: *Direct Flow*, green; *Retained Inflow*, yellow; *Delayed Ejection Flow*, blue; *Residual Volume*, red. LA, Left Atrium; LV, Left Ventricle.

The flow data from each of the nine subjects (Table 1) were analyzed twice by Inv 1 and once by Inv 2, using the proposed approach, resulting in pathline visualization of the four LV flow components, as demonstrated in Figures 1 and 2 and Additional file 1.

**Figure 3:**
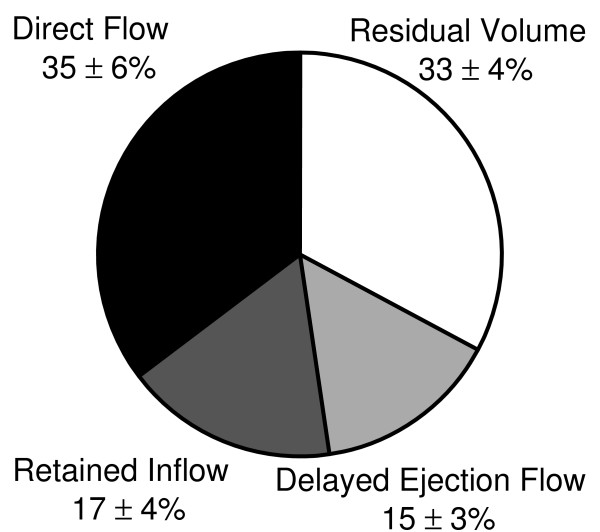
**Pie Chart**. Illustration of the four LV flow components as a percentage of LV end-diastolic volume (mean ± SD) in the six healthy subjects, obtained by investigator 1 analysis 1.

The relative size of the four flow components of intra-ventricular flow in the six healthy subjects is shown in Figure 3. The two largest components were *Direct Flow *and *Residual Volume*.

The LV outflow, computed from 3D cine PC-CMR obtained by Inv1 A1, was related to LV outflow quantified using 2D cine through-plane PC-CMR and Doppler ultrasound, respectively, for all nine subjects (Table 2). Outflow estimates obtained from 2D cine through-plane PC-CMR appeared to be larger, while outflow estimates from Doppler Ultrasound appeared to be smaller.

**Table 2:** LV outflow volume measured with the proposed method and other CMR and echocardiographic techniques.

Subject	Outflow, 3D cine PC-CMR (mL)	Outflow, 2D cine through-plane PC-CMR (mL)	Outflow, Doppler Echo, (mL)
1 Healthy	53	48	50
2 Healthy	92	105	75
3 Healthy	59	74	48
4 Healthy	73	72	62
5 Healthy	71	84	53
6 Healthy	61	74	51
7 DCM	59	71	66
8 DCM	80	95	68
9 DCM	58	72	46
Mean ± SD	67 ± 13	77 ± 16	58 ± 10

DCM, dilated cardiomyopathy; PC, phase-contrast. 3D cine PC-CMR measurements are from Investigator I, Analysis 1.

As a test of the accuracy of the method proposed, the LV inflow and outflow volumes were compared. No significant difference between inflow and outflow volume was found in any of the three analyses (Table 3).

**Table 3:** LV inflow and outflow based on 4D flow data from the three different analyses

	Investigator 1 (analysis 1)	Investigator 1 (analysis 2)	Investigator 2
Inflow	68 ± 11	71 ± 9	72 ± 12
Outflow	67 ± 13	69 ± 13	70 ± 15
P-value	NS	NS	NS

Mean ± SD for all 9 subjects.

**Figure 4:**
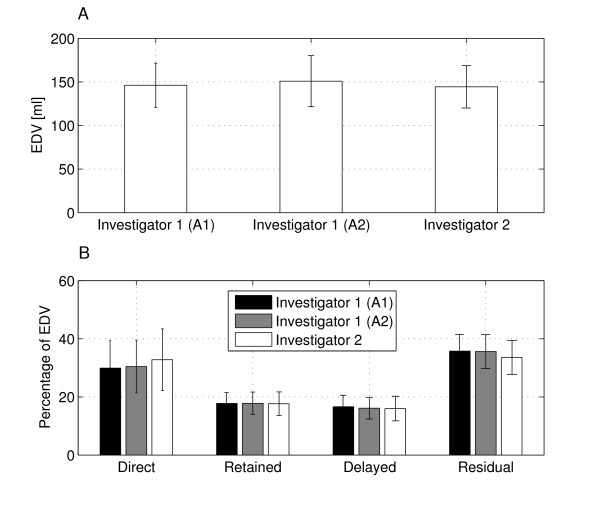
**EDV and intra-ventricular components**. LV end-diastolic volume (EDV) (A), and the four LV flow components as a percentage of EDV (B), from the three different analyses. Mean ± SD for all 9 subjects. A1, analysis 1; A2, analysis 2.

The inter- and intra-observer variability was analyzed for the EDV and the four intra-ventricular flow components (Figure 4). As shown in Table 4 no significant differences were found for any of the parameters investigated. The end-diastolic volume comparison between Inv 1 A2 and Inv 2 resulted in borderline significance.

**Table 4:** Inter- and intra-observer variability of the LVEDV and the four LV flow components as a percentage of EDV

	I1 A1	P-value vs I1 A2	I1 A2	P-value vs I2	I2	P-value vs I1 A1
EDV	146 ± 25	NS	151 ± 29	0.010	144 ± 24	NS
Direct flow	30 ± 10%	NS	30 ± 9%	NS	33 ± 11%	NS
Retained inflow	18 ± 4%	NS	18 ± 4%	NS	18 ± 4%	NS
Delayed ejection flow	17 ± 4%	NS	16 ± 4%	NS	16 ± 4%	NS
Residual volume	36 ± 6%	NS	36 ± 6%	NS	33 ± 6%	NS

Mean ± SD for all 9 subjects. P-values < 0.01 are considered statistically significant. EDV, End diastolic volume; NS, Not Significant; I1 A1, Investigator 1 Analysis 1; I1 A2, Investigator 1 Analysis 2; I2; Investigator 2.

## Discussion

In the present study, a novel analysis approach for 4D intra-ventricular flow data was developed and validated. The proposed analysis approach: (1) allowed quantification of the volume, distribution and timing of separate components of LV flow in all nine data sets; (2) obtained a measurement of the LV outflow volume that appeared to be in between the magnitudes obtained by 2D cine through-plane PC-CMR and Doppler ultrasound, respectively; (3) achieved equal magnitudes of the LV inflow and outflow volumes; and (4) showed a high intra- and inter-observer reproducibility for the assessment of the volumes of the LV flow components. In addition, the present analysis approach was both less user-dependent and time-consuming than our previous technique: using the present approach the time required for each analysis was reduced from hours per analysis to minutes. In fact, the only user interaction necessary is the segmentation of the LV from short axis morphological images and to determine the times of IVC and IVR in the 3D cine PC-CMR data.

The proposed approach enables the quantification and extended visualization (Additional file 1) of the flow-derived LVEDV separated into the four intra-ventricular flow components (*Direct Flow*, *Retained Inflow*, *Delayed Ejection Flow *and *Residual Volume*). The visualization is extended because this approach facilitates the visualization of the residual volume and the other flow components over one cardiac cycle. When comparing the present measurements of these flow components with our previous findings [22], some differences were observed, such as a higher amount of the *Direct Flow *and *Residual Volume *using the present approach. We believe that these differences may be explained by a number of factors. The present velocity data have higher spatial and temporal resolution, and the previous data were acquired without respiratory compensation. The continuous emission of pathlines over diastolic filling might lead to an underestimation of inflow using our previous technique. The previous technique approximated the EDV by making a wire frame hull of the particle trace positions at end diastole; this likely caused an underestimation of the true EDV. The *Residual Volume *as estimated by the previous method used this inaccurate EDV; the volume was not directly visualized using pathlines. In contrast, the present analysis approach allows tracing of the *Residual Volume *as well as the other flow components. There is a risk of overestimating this component in the presented approach, since the limit of the LV cavity is defined as the compact myocardium. It is possible that some of the IVC traces are emitted from the trabeculae but are included in the IVR blood volume. However, in both the previous and present analysis approach, this would constitute a consistent error as it is done in the same way in every data set.

The LV outflow volume measured with the proposed approach correlated with measurements obtained with both 2D cine through-plane PC-CMR and Doppler ultrasound (Table 2). The flow-based volume appeared to fall in between the volumes obtained from the two other methods. LV stroke volumes obtained by ultrasound are generally smaller than those obtained by CMR. Stalder et al [26] compared 3D cine PC-CMR and 2D cine PC-CMR and found that 3D cine PC-CMR gave slightly lower velocities. This is most likely due to lower spatial resolution in the 3D cine PC-CMR data. Although the stroke volume and end-diastolic volume can be measured using the proposed analysis, we do not suggest that it should replace 2D cine through-plane PC-CMR or ultrasound techniques for the assessment of these parameters. The strength of the method proposed is instead that each part of the LV blood volume is monitored throughout the cardiac cycle, enabling assessment of the dynamic and multidimensional blood flow organization within the beating LV.

We validated the accuracy of this approach by comparing LV inflow and outflow volumes in each subject. We have not validated all different components and the blood flow patterns in the LV. However, LV inflow consists of *Direct Flow *and *Retained Inflow*, the LV outflow consists of *Direct Flow *and *Delayed Ejection Flow*. The *Residual Volume *is not validated, but it is based on the same flow data and segmentation of the LV. The segmentation of the LV from short axis morphological images is well validated [27,28] and often part of the clinical CMR protocol. Visualization of blood flow using pathlines has been used extensively before [15,16,18-22]. The accuracy of the computed pathlines depends, however, on a large number of factors, including temporal and spatial resolution, VENC, field strength, CMR hardware, and intravoxel dephasing due to turbulence. We recommend therefore performing the validation based on inflow vs outflow for every patient study using this analysis approach, as it is easy to perform without any extra acquisitions or analysis.

Inter- and intra-observer variability was determined for flow-based EDV and the four intra-ventricular flow components (Table 4 and Figure 4). No significant differences between observers were detected; the difference in EDV obtained by Inv1 A2 and Inv 2 were of borderline significance. We believe that, of all the steps of the analysis, the LV segmentation has the most important impact on the reproducibility of the results. This is especially true for the selection of the most basal short axis slice in the LV, as this plane is used to determine whether the traces leave the LV in the basal-apical direction. We choose this plane in order to avoid the inclusion of the LA volume into the LV segmentation, and in most cases its position was easy to identify. In terms of other regions of the LV, an overestimation of the ventricular volume will most likely give an overestimation of the *Residual Volume*, while an underestimation may remove important portions of the flowing blood. In our segmentation protocol we chose to segment compact myocardium. We preferred a clinically useful segmentation approach that minimizes the risk of missing important flow due to partial volume effects; as the slice thickness was 8 mm it is very likely that the structure of the endocardial border varies through the plane, and that the pixel value is an average value of the tissue in the pixel. There is no consensus in the literature regarding the impact of including trabeculae and papillary muscles in the ventricular volume. Papavassiliu et al [27] concluded that even though the trabeculae significantly influence the LV volume, the superior reproducibility of their inclusion in the LV volume makes this approach most appropriate for clinical use. Sievers et al [28] concluded that, although there are systematic differences between inclusion and exclusion of trabeculae and papillary muscles, these differences are small and do not necessarily have a clinical relevance in healthy or diseased hearts and that inclusion is significantly faster. Also in the 3D cine PC-CMR data, voxels partially covered by trabeculae will suffer from partial volume effects, leading to an underestimation of the velocity of the flowing blood in these voxels. Another factor interactively set by the investigator is timing, but this factor is less critical than the LV segmentation procedure as long as particles are emitted at a time frame when no flow is crossing the mitral or aortic valve.

3D cine PC-CMR flow data comprise large amounts of information and the ability to quantify important aspects of these data with a user-friendly and accurate tool opens the door to larger studies which could address unanswered physiological and clinical questions. In addition to the assessment of the volume and distribution of the different LV flow components, the proposed analysis approach also allows quantification of the kinetic energy change of these components throughout the cardiac cycle. Recent findings in normal hearts suggest that the *Direct Flow *best preserves its kinetic energy during transit through the LV, while the *Retained Inflow *decelerates and requires greater addition of energy to achieve eventual ejection from the chamber [22]. In dilated and hypocontractile hearts, preliminary findings have shown that the normal organization of LV flow is altered, and that this abnormal flow pattern affects pumping efficiency. The decreased direct flow and increased residual volume seen in these patients may be disadvantageous from an LV pumping efficiency point of view [22]. The ability to measure ventricular performance using multidimensional blood flow analysis creates the potential for new perspectives on pathophysiology, early diagnosis, treatment options, and prognosis.

### Limitations

Although no significant differences between the LV inflow and outflow were detected for any of the three analyzes, in a few subjects larger differences were observed. Careful review of these cases suggested that the morphological and flow data did not match exactly, so that the morphological data were displaced relative to the flow data. The reason for this was likely the result of subject motion between the acquisition of the 3D cine PC-CMR and the bSSFP data. This error could be minimized by careful instruction of the patient not to move between the scans. A more extensive approach may be to use volume registration. This will be further investigated in future studies.

Aortic or mitral valve insufficiency is at present a limitation because the regurgitant pathlines will be inaccurately included in the volume of the components that leave the LV. However, regurgitant flow pathlines will be identifiable using the timing and location where they leave the segmented volume. Detection and correction for these regurgitant pathlines will be implemented and evaluated for studies including patients with valve insufficiency. In the present study, valvular regurgitation that was more than trace constituted an exclusion criterion.

## Conclusions

A semi-automatic analysis approach for the quantification and visualization of 4D LV flow data was developed and validated in normal and dilated, myopathic hearts. The presented technique, utilizing the integration of morphological data and 3D cine PC-CMR flow data, allows for quantification of the volume, distribution and timing of separate components of LV flow and extends visualization of LV flow. The analysis approach was shown to yield accurate LV inflow and outflow volumes, and a high intra- and inter-observer reproducibility for the assessment of the flow components, which may open the door to larger clinical studies.

## List of abbreviations

AoV: aortic valve; DCM: dilated cardiomyopathy; EF: ejection fraction; IVC: Isovolumetric contraction; IVR: Isovolumetric relaxation; MV: Mitral Valve; LA: Left Atrium; LV: Left Ventricle; PC-CMR: Phase Contrast Cardiovascular Magnetic Resonance; SD: Standard Deviation.

## Competing interests

The authors declare that they have no competing interests.

## Authors' contributions

JE, CJC, TE, PD and AFB have been involved in conception and design. CJC recruited the subjects. CJC and PD have been involved in data acquisition. PD developed post processing tools. PD and JE post processed the data. JE implemented and evaluated the method along the implementation. JE, CJC, PD, JEN, AFB and TE analyzed and interpreted the data. JE and CJC performed the statistical analysis. TE and CJC supervised the study. JE and CJC drafted the manuscript. CJC, PD, JEN, AFB and TE critically revised the manuscript. All authors had full access to the data and take responsibility for its integrity. All authors have read and agree to the manuscript as written.

## Supplementary Material

Additional file 1 **Left heart flow in a healthy subject**. Movie showing left heart intra cardiac blood flow in a healthy 61 year old male, all four components visualized with pathlines and a semi-transparent 3-chamber image to provide morphological orientation. The pathlines are color coded according to: *Direct Flow*, green; *Retained Inflow*, yellow; *Delayed Ejection Flow*, blue; *Residual Volume*, red. LA, Left Atrium; LV, Left Ventricle. The file-format is MPEG, which may be opened using standard software. Click here for file
